# Trends of drug expenditure in Taiwan National Health Insurance before and during COVID-19 pandemic

**DOI:** 10.3389/fmed.2024.1388569

**Published:** 2024-08-20

**Authors:** Shu-I Wu, An-Sheng Lee, Ching-Hu Chung

**Affiliations:** ^1^Department of Medicine, Mackay Medical College, Taipei City, Taiwan; ^2^Department of Psychiatry, Mackay Memorial Hospital, Taipei City, Taiwan; ^3^Suicide Prevention Center, Mackay Memorial Hospital, Taipei City, Taiwan

**Keywords:** drug expenditure, medical center, regional hospital, district hospital, clinics, COVID-19 pandemic

## Abstract

**Background:**

The Taiwanese government adopted the National Health Insurance (NHI) system in March 1995. This study aimed to understand the difference in medication costs before (year 2019) and during the COVID-19 pandemic (2020–2021) among different hospitals for treating their patients.

**Methods:**

The NHI claims database consisting of claims of prescription drugs for inpatients (IPD) and outpatients (OPD) in Taiwan was used to determine drug expenditure in different hospitals, particularly the top 10 prescription Anatomical Therapeutic Chemical (ATC) categories.

**Results:**

In medical centers, L01X (other antineoplastic agents) showed the highest drug expenditure, followed by L04A (immunosuppressants) and J05A (direct-acting antivirals). The drug expenditure pattern in regional hospitals was similar to that in medical centers, with L01X (other antineoplastic agents) showing the highest drug expenditure. L01X (other antineoplastic agents) also showed the highest drug expenditure in district hospitals, followed by N05A (antipsychotics) and A10B (blood glucose-lowering drugs, excluding insulin). In clinics, A10B (blood glucose-lowering drugs, excluding insulin) showed the highest drug expenditure. The total medication costs in 2021 were lower or similar to those in 2019. The use of systemic use anti-infectives decreased over time in OPDs among all hospita1 levels but increased in IPDs in medical centers and district hospitals. Furthermore, our analysis revealed that the trend in drug expenditure closely mirrored the trend in drug prescription volume for the highest annual sum cost item among the top 10 drug subgroups across different hospital levels.

**Conclusion:**

Our analysis found that annual drug expenditures in 2021 were lower or similar to those in 2019, suggesting that the COVID-19 pandemic has contributed to this reduction in drug expenditure.

## Introduction

The National Health Insurance (NHI) system in Taiwan is a comprehensive and government-run healthcare system that provides affordable and accessible medical services to the residents of Taiwan. Established in 1995, the NHI is designed to ensure that all citizens have access to high-quality healthcare without facing financial barriers (1). The NHI nearly covers the entire population of Taiwan, including citizens, residents, and foreigners with legal residency. Enrollment in the program is mandatory for all eligible individuals (2). The NHI offers various medical services, including outpatient and inpatient care, preventive services, dental care, prescription medications, and traditional Chinese medicine. The coronavirus disease 2019 (COVID-19) pandemic has placed significant strain on healthcare systems worldwide. In Taiwan, an increase in the demand for healthcare services, including testing, treatment, and vaccination, would likely have occurred (3). This increased demand could have put pressure on the NHI in terms of resource allocation and managing the costs associated with the pandemic response.

Global health systems were profoundly shaken by the unexpected onset of the COVID-19 pandemic, challenging their resilience and adaptability to sustain essential functions (4). Assessing the effectiveness of health systems in shielding households from financial burdens arising from healthcare expenses during this crisis is crucial. The impact of COVID-19 on drug prescriptions is multifaceted and varies across regions and healthcare systems. With lockdowns and social-distancing measures in place, a significant increase in the use of telemedicine and remote consultations has been observed (5). This has affected the volume of prescriptions made because drug delivery may be an issue for the healthcare provider’s prescription. The pandemic has also led to shifts in drug usage patterns. Some medications, such as those used to treat respiratory symptoms or complications associated with COVID-19, have seen increased demand (6, 7). In contrast, disruptions in the supply chain for certain drugs have been observed, leading to shortages in some regions. Because of lockdowns and concerns about virus transmission, some individuals have delayed seeking medical care for nonurgent issues. This delay may have influenced the prescription of medications for chronic conditions because patients may have been managing their conditions differently during the pandemic (8).

Our previous study indicated that annual drug expenditures increased across all hospitals from 2016 to 2018 (9). However, the impact of COVID-19 on patient willingness to visit clinics and overall drug expenditures remains unclear. To evaluate the practical implications and address the existing knowledge deficit regarding the impact of COVID-19 on drug prescriptions (including drug expenditure and volume), we leveraged the NHI claims database encompassing all prescribed medications in Taiwan. Our analysis aimed to discern the patterns in annual drug costs across different hospital levels by identifying the top 10 prescription categories based on the Anatomical Therapeutic Chemical (ATC) classification system, focusing on those with the highest annual prescription drug costs. We also examined the drug prescription volume trend for the highest drug expenditure item among these top 10 drug subgroups.

## Materials and methods

### Data source

This retrospective cohort study leveraged claims records from 1 million patients from the NHI database from 2019 to 2021. The comprehensive claims database encompasses original data on drug prescriptions for all beneficiaries in Taiwan. Using the ATC classification, we computed specific drug prescription categories (10). Hospital levels were identified through the registry for contracted medical facilities. This study enrolled all hospitals in Taiwan, comprising 22,992, 23,132, and 23,278 hospitals in 2019, 2020, and 2021, respectively (11). Notably, all drug prescriptions within the Taiwan NHI were included, and no selection criteria were applied.

### Assessment

The main variable under observation was the declared amount (annual sum cost of specific prescription drugs within the Taiwan NHI) of specific prescription drugs across various hospital levels. Hospital levels were categorized into four types: medical centers, regional hospitals, district hospitals, and clinics/others. Fifteen prescription drug types were considered, spanning categories including (A) alimentary tract and metabolism; (B) blood and blood-forming organs; (C) cardiovascular system; (D) dermatologicals; (G) genitourinary system and sex hormones; (H) systemic hormonal preparations excluding sex hormones and insulins; (J) anti-infectives for systemic use; (L) antineoplastic and immunomodulating agents; (M) musculoskeletal system; (N) nervous system; (P) antiparasitic products, insecticides, and repellents; (R) respiratory system; (S) sensory organs; (V) various; and (U) others (with missing ATC coding). The top 10 highest drug annual sum cost ATC categories for different hospital levels were identified based on the annual sum cost of prescription drugs belonging to the same 3rd ATC level (pharmacological subgroup, form A01A to V10X). Furthermore, we divided drug expenditure (annual sum cost of prescription drugs within the Taiwan NHI) into IPD and OPD. The currency exchange rates at the end of the index year were 29.983 in 2019, 28.095 in 2020, and 27.699 in 2021, with the NTD to USD ratio fixed at 1:29 in this study. The unit of cost presented in the Tables 1–4 is reported in millions of USD. The median annual drug cost in 2019–2021 was used to present the drug expenditure in the index ATC group or selected drug. The drug prescription volume trend for the highest drug expenditure item among the top 10 drug subgroups across different hospital levels was measured using the sum prescription quantity.

### Data analyses

SAS 9.4 (SAS Institute Inc., Cary, NC) was used for data analyses. The selected ATC drug groups or prescription drugs were used to describe their annual sum cost or sum prescription quantity among different hospital levels during 2019–2021.

## Results

Among all original claims encompassing drug prescription data for beneficiaries, the annual drug expenditures in medical centers for the 15 specified types rose from $2,511.12 million in 2019 to $2,621.35 million in 2020 (4.38% incremental) and decreased to $2,549.59 million in 2021 (2.74% decremental) (Table 1). Correspondingly, expenditures in regional hospitals slightly increased from $1,920.44 million to $1,920.49 million; in district hospitals, expenditures increased from $710.52 million to $757.30 million (6.58% incremental); and in clinics/others, expenditures increased from $518.06 million to $531.50 million (2.59% incremental) during 2019–2020. In contrast, expenditures in regional hospitals decreased from $1,920.49 million to $1,741.99 million (9.29% decremental); in district hospitals, expenditures decreased from $757.30 million to $701.26 million (7.40% decremental); and in clinics/others, expenditures decreased from $531.50 million to $373.10 million (29.80% decremental) during 2020–2021.

**Table 1:** Drug expenditure among different levels of hospital.

	IPD			OPD			Total		
	2019	2020	2021	2019	2020	2021	2019	2020	2021
Medical center	510.17	532.83 (+4.44%)	522.98 (−1.85%)	2000.95	2088.53 (+4.38%)	2026.61 (−2.96%)	2511.12	2621.35 (+4.39%)	2549.59 (−2.74%)
Regional hospital	377.61	382.62 (+1.33%)	371.15 (−3.00%)	1542.83	1537.86 (−0.32%)	1370.84 (−10.86%)	1920.44	1920.49 (0.00%)	1741.99 (−9.29%)
District hospital	111.96	124.68 (+11.36%)	137.49 (+10.27%)	598.56	632.61 (+5.69%)	563.77 (−10.88%)	710.52	757.30 (+6.58%)	701.26 (−7.40%)
Clinics/others	0.61	0.56 (−8.49%)	0.56 (−0.65%)	517.45	530.94 (+2.61%)	372.54 (−29.83%)	518.06	531.50 (+2.59%)	373.10 (−29.8%)

Hospital levels were categorized into four types: medical centers, regional hospitals, district hospitals, and clinics/others. We divided drug expenditure (annual sum cost of prescription drug within the Taiwan NHI) into in-patient department (IPD) and outpatient department (OPD). The drug expenditure was indicated by million USD and the exchange rate used in this study was 1:29. (+ − percentage) indicated the percent change as compared with the previous year.

Table 2 presents the 15 categories of annual drug expenditures. The median annual drug cost in 2019–2021 was used to present the drug expenditure in the index ATC group or selected drug. Within medical centers, the highest overall drug expenditure (including IPD and OPD) was observed in antineoplastic and immunomodulating agents ($1027.48 million/year (median) with 14.15% incremental in 2020 and 10.42% incremental in 2021), followed by anti-infectives for systemic use ($409.64 million/year (median) with 7.74% decremental in 2020 and 3.89% decremental in 2021), alimentary tract and metabolism ($296.35 million/year (median) with 2.73% incremental in 2020 and 1.10% incremental in 2021), and blood disorders ($261.70 million/year (median) with 2.97% incremental in 2020 and 1.11% incremental in 2021). The drug expenditure pattern in regional hospitals mirrored that of medical centers, with antitumor and immunological agents ($537.70 million/year (median) with 14.67% incremental in 2020 and 10.95% incremental in 2021) exhibiting the highest overall drug expenditure, followed by anti-infectives for systemic use ($343.97 million/year (median) with 14.96% decremental in 2020 and 12.67% decremental in 2021), alimentary tract and metabolism ($209.55 million/year (median) with 0.80% decremental in 2020 and 1.07% decremental in 2021), and blood disorders ($192.54 million/year (median) with 1.54% incremental in 2020 and 1.51% decremental in 2021). In district hospitals, the highest overall drug expenditure was on anti-infectives for systemic use ($130.74 million/year (median) with 4.37% decremental in 2020 and 9.68% decremental in 2021), followed by alimentary tract and metabolism ($116.33 million/year (median) with 6.62% incremental in 2020 and 4.33% incremental in 2021), cardiac and hypertension ($113.33 million/year with 3.38% incremental in 2020 and 2.98% incremental in 2021), and nervous system ($102.05 million/year (median) with 7.16% incremental in 2020 and 6.52% incremental in 2021). Meanwhile, in clinics/others, the highest overall drug expenditure was associated with cardiac and hypertension ($162.75 million/year with 6.69% incremental in 2020 and 6.21% incremental in 2021), followed by alimentary tract and metabolism ($114.57 million/year with 8.01% incremental in 2020 and 7.57% incremental in 2021), nervous system ($75.51 million/year (median) with 6.62% incremental in 2020 and 6.07% incremental in 2021), and respiratory system ($45.16 million/year with 15.24% decremental in 2020 and 12.02% decremental in 2021).

**Table 2:** Drug expenditure among different level of hospitals based on 15 categories of the first level Anatomical Therapeutic Chemical code.

Category	IPD			OPD			Total		
	2019	2020	2021	2019	2020	2021	2019	2020	2021
(A) Medical center									
A	42.92	42.71 (−0.48%)	37.44 (−12.36%)	245.55	253.69 (+3.29%)	262.17 (+3.36%)	288.47	296.35 (+2.73%)	299.60 (+1.10%)
B	60.90	59.59 (−2.16%)	55.25 (−7.28%)	193.24	202.11 (+4.59%)	209.35 (+3.58%)	254.14	261.7 (−2.97%)	264.61 (+1.11%)
C	15.43	15.14 (−1.88%)	14.88 (−1.70%)	177.44	173.54 (−2.20%)	169.25 (−2.47%)	192.86	188.68 (−2.17%)	184.13 (−2.41%)
D	1.37	1.30 (−5.12%)	1.23 (−5.13%)	9.56	10.09 (+5.51%)	12.21 (+21.01%)	10.93	11.39 (+4.18%)	13.44 (+18.03%)
G	0.98	0.96 (−2.34%)	0.98 (−1.85%)	32.26	30.75 (−4.68%)	30.92 (+0.55%)	33.24	31.71 (−4.61%)	31.90 (+0.59%)
H	7.42	7.04 (−5.21%)	7.07 (+0.42%)	33.07	33.21 (+0.42%)	32.42 (−2.37%)	40.49	40.24 (−0.61%)	39.49 (−1.88%)
J	194.29	196.28 (+1.02%)	202.76 (+3.30%)	249.70	213.36 (−14.55%)	190.93 (−10.52%)	443.99	409.64 (−7.74%)	393.69 (−3.89%)
L	144.96	164.93 (+13.78%)	157.02 (−4.80%)	755.16	862.55 (+14.22%)	977.52 (13.33%)	900.12	1027.48 (+14.15%)	1134.54 (+10.42%)
M	4.72	11.38 (+141.13%)	13.59 (+19.35%)	46.93	46.52 (−0.87%)	48.18 (+3.57%)	51.65	57.9 (+12.11%)	61.77 (+6.67%)
N	24.14	24.68 (+2.26%)	24.45 (−0.95%)	145.50	147.69 (+1.51%)	145.92 (−1.20%)	169.64	172.38 (+1.61%)	170.37 (−1.17%)
P	0.08	0.07 (−12.06%)	0.08 (+15.61%)	1.34	1.33 (−0.73%)	1.25 (−5.98%)	1.42	1.40 (−1.35%)	1.33 (−4.93%)
R	5.81	4.93 (−15.06%)	4.84 (−1.98%)	48.62	48.53 (−0.18%)	49.95 (−2.93%)	54.43	53.47 (−1.77%)	54.79 (+2.47%)
S	0.47	0.46 (−2.73%)	0.44 (−3.70%)	42.11	42.57 (+1.09%)	42.85 (+0.66%)	42.58	43.02 (−1.05%)	43.29 (+0.61%)
V	3.03	3.11 (+2.61%)	2.89 (−7.08%)	20.19	21.78 (+7.86%)	21.95 (+0.77%)	23.23	24.90 (−7.18%)	24.84 (−0.21%)
U	3.65	0.25 (−93.22%)	0.07 (−72.46%)	0.27	0.84 (+208.14%)	0.99 (+17.86%)	3.92	1.09 (−72.29%)	1.06 (−2.71%)
(B) Regional hospital									
A	19.22	19.30 (0.42%)	18.87 (−2.25%)	192.03	190.25 (−0.93%)	188.44 (−0.95%)	211.25	209.55 (−0.80%)	207.30 (−1.07%)
B	41.77	41.64 (−0.32%)	41.75 (+0.27%)	150.76	153.86 (+2.05%)	150.79 (−2.00%)	192.54	195.5 (−1.54%)	192.54 (−1.51%)
C	11.57	11.33 (−2.07%)	11.47 (+1.23%)	194.69	187.42 (−3.74%)	181.37 (−3.23%)	206.27	198.75 (−3.64%)	192.84 (−2.97%)
D	1.18	1.15 (−2.59%)	1.11 (−3.48%)	9.27	9.39 (+1.28%)	9.73 (+3.63%)	10.45	10.54 (+0.85%)	10.84 (+2.86%)
G	1.03	1.02 (−0.46%)	1.01 (−1.35%)	34.17	32.09 (−6.09%)	32.09 (−0.02%)	35.2	33.11 (−5.93%)	33.09 (−0.06%)
H	5.87	5.37 (−8.59%)	5.13 (−4.46%)	19.10	18.96 (−0.72%)	18.25 (−3.72%)	24.97	24.33 (−2.57%)	23.38 (−3.88%)
J	145.20	141.77 (−2.36%)	139.13 (−1.87%)	259.26	202.19 (−22.01%)	161.26 (−20.24%)	404.46	343.97 (−14.96%)	300.39 (−12.67%)
L	108.03	119.25 (−10.38%)	111.48 (−6.51%)	360.87	418.45 (+15.96%)	485.12 (+15.93%)	468.9	537.70 (+14.67%)	596.60 (+10.95%)
M	3.83	3.49 (−8.93%)	3.35 (−3.99%)	54.95	53.27 (−3.04%)	52.71 (−1.06%)	58.77	56.76 (−3.43%)	56.06 (−1.24%)
N	28.96	29.59 (+2.14%)	29.57 (−0.06%)	168.53	173.91 (+3.19%)	174.97 (+0.61%)	197.5	203.49 (+3.04%)	204.53 (+0.51%)
P	0.12	0.11 (−3.75%)	0.11 (−5.93%)	1.10	1.08 (−1.61%)	0.98 (−9.56%)	1.22	1.19 (−1.82%)	1.08 (−9.21%)
R	7.46	6.076 (−18.52%)	5.62 (−7.54%)	58.82	56.95 (−3.17%)	55.57 (−2.43%)	66.27	63.03 (−4.90%)	61.18 (−2.92%)
S	0.25	0.22 (−10.37%)	0.21 (−5.72%)	29.29	28.95 (−1.16%)	29.04 (+0.31%)	29.54	29.17 (−1.24%)	29.25 (+0.27%)
V	2.26	2.31 (+2.07%)	2.34 (+1.37%)	9.98	11.09 (+11.13%)	11.90 (+7.32%)	12.24	13.39 (−9.45%)	14.24 (+6.29%)
U	0.86	0.00 (−99.82%)	0.02 (+1080%)	0.02	0.00 (−89.49%)	0.00 (+35.54%)	0.88	0.00 (−99.60%)	0.02 (477.38%)
(C) District hospital									
A	6.53	7.33 (+12.15%)	7.47 (+1.89%)	102.57	109.00 (+6.27%)	113.91 (+4.50%)	109.1	116.33 (+6.62%)	121.37 (+4.33%)
B	11.99	12.01 (+0.19%)	12.96 (+7.88%)	57.81	56.19 (−2.81%)	58.77 (+4.59%)	69.8	68.20 (−2.29%)	71.73 (+5.17%)
C	3.57	3.77 (+5.58%)	4.03 (+6.96%)	104.13	107.56 (+3.30%)	110.62 (+2.84%)	107.7	111.33 (+3.38%)	114.65 (+2.98%)
D	0.46	0.51 (+9.83%)	0.54 (+6.26%)	5.52	5.69 (+3.13%)	6.03 (+6.04%)	5.98	6.20 (+3.64%)	6.57 (+6.06%)
G	0.53	0.54 (+3.15%)	0.53 (−2.60%)	19.00	19.42 (+2.19%)	20.87 (+7.50%)	19.53	19.96 (+2.21%)	21.4 (+7.23%)
H	1.73	1.63 (−5.63%)	1.68 (+2.59%)	8.74	9.50 (+8.69%)	9.77 (+2.83%)	10.47	11.13 (+6.32%)	11.44 (+2.80%)
J	53.30	56.68 (+6.35%)	62.90 (+10.96%)	83.41	74.06 (−11.21%)	55.19 (−25.48%)	136.71	130.74 (−4.37%)	118.08 (−9.68%)
L	11.09	18.78 (+69.39%)	22.80 (+21.44%)	60.82	86.67 (+42.51%)	124.96 (+44.18%)	71.9	105.45 (+46.65%)	147.76 (+40.13%)
M	0.96	1.10 (+14.30%)	1.21 (+10.37%)	33.34	34.05 (+2.14%)	35.68 (+4.78%)	34.3	35.15 (+2.48%)	36.89 (+4.95%)
N	17.73	18.54 (+4.58%)	19.52 (+5.27%)	77.50	83.51 (+7.75%)	89.18 (+6.80%)	95.23	102.05 (+7.16%)	108.70 (+6.52%)
P	0.08	0.08 (+0.16%)	0.09 (+17.32%)	0.61	0.65 (+5.80%)	0.65 (+0.20%)	0.69	0.72 (+5.19%)	0.74 (+1.98%)
R	3.61	3.23 (−10.47%)	3.25 (+0.57%)	31.97	31.85 (−0.36%)	32.65 (+2.49%)	35.58	35.08 (−1.39%)	35.89 (+2.31%)
S	0.08	0.09 (+13.48%)	0.08 (−11.44%)	10.69	11.71 (+9.54%)	12.92 (+10.36%)	10.77	11.80 (+9.57%)	13.00 (+10.20%)
V	0.32	0.40 (+27.88%)	0.45 (+11.70%)	2.46	2.76 (12.16%)	3.20 (+15.84%)	2.78	3.16 (+13.95%)	3.65 (+15.31%)
U	0.00	0.00 (−100.00%)	0.00 (−)	0.00	0.00 (−)	0.00 (+131.93%)	0.00	0.00 (−97.63%)	0.00 (+135.13%)
(D) Clinics/others									
A	0.02	0.02 (−2.29%)	0.02 (+9.77%)	106.05	114.55 (+8.01%)	123.22 (+7.57%)	106.07	114.57 (+8.01%)	123.24 (+7.57%)
B	0.24	0.23 (−4.54%)	0.22 (−1.66%)	13.03	13.73 (+5.35%)	15.45 (+12.30%)	13.27	13.95 (+5.17%)	15.68 (+12.37%)
C	0.01	0.01 (−4.46%)	0.01 (−7.28%)	152.53	162.74 (+6.69%)	172.85 (+6.21%)	152.54	162.75 (+6.69%)	172.85 (+6.21%)
D	0.00	0.00 (+0.72%)	0.00 (+22.74%)	14.38	15.05 (+4.66%)	15.03 (−0.13%)	14.38	15.05 (+4.66%)	15.03 (−0.13%)
G	0.07	0.07 (+4.29%)	0.08 (+9.59%)	12.67	14.04 (+10.08%)	15.24 (+8.53%)	12.74	14.11 (+10.77%)	15.32 (+8.53%)
H	0.05	0.05 (−2.58%)	0.05 (+4.22%)	2.93	3.08 (+5.22%)	3.17 (+2.93%)	2.97	3.12 (+5.10%)	3.22 (+2.95%)
J	0.16	0.13 (−14.88%)	0.13 (−0.14%)	43.85	39.92 (−8.96%)	33.77 (−15.39%)	44.01	40.05 (−8.98%)	33.91 (−15.34%)
L	0.00	0.00 (+196.67%)	0.00 (−100.00%)	1.02	1.08 (+5.98%)	1.12 (+4.27%)	1.02	1.08 (+5.98%)	1.12 (+4.27%)
M	0.03	0.03 (−5.30%)	0.03 (+8.16%)	23.94	22.44 (−6.28%)	21.81 (−2.80%)	23.97	22.47 (−6.28%)	21.84 (−2.79%)
N	0.03	0.02 (−42.29%)	0.01 (−66.45%)	70.79	75.49 (+6.64%)	80.09 (+6.09%)	70.82	75.51 (+6.62%)	80.09 (+6.07%)
P	0.00	0.00 (−14.85%)	0.00 (+35.04%)	0.47	0.47 (+0.21%)	0.46 (−3.53%)	0.47	0.47 (+0.20%)	0.46 (−3.52%)
R	0.01	0.00 (−58.93%)	0.00 (−45.60%)	53.27	45.16 (−15.23%)	39.73 (−12.02%)	53.28	45.16 (−15.24%)	39.73 (−12.02%)
S	0.00	0.00 (−59.62%)	0.00 (+142.86%)	21.39	22.25 (+4.03%)	22.56 (+1.39%)	21.39	22.25 (+4.03%)	22.56 (+1.39%)
V	0.00	0.00 (−15.16%)	0.00 (−10.26%)	1.13	0.95 (−16.17%)	0.88 (−7.44%)	1.13	0.95 (−16.17%)	0.88 (−7.44%)
U	0.00	0.00 (−)	0.00 (−)	0.00	0.00 +(1299.10%)	0.01 (+427.56%)	0.00	0.00 (+1299.10%)	0.01 (+427.56%)

Hospital levels were categorized into four types: medical centers, regional hospitals, district hospitals, and clinics/others. We divided drug expenditure (annual sum cost of prescription drug within the Taiwan NHI) into in-patient department (IPD) and outpatient department (OPD). Fifteen prescription drug types were considered, spanning categories including (A) alimentary tract and metabolism; (B) blood and blood-forming organs; (C) cardiovascular system; (D) dermatologicals; (G) genitourinary system and sex hormones; (H) systemic hormonal preparations excluding sex hormones and insulins; (J) anti-infectives for systemic use; (L) antineoplastic and immunomodulating agents; (M) musculoskeletal system; (N) nervous system; (P) antiparasitic products, insecticides, and repellents; (R) respiratory system; (S) sensory organs; (V) various; and (U) others (with missing ATC coding). The drug expenditure was indicated by million USD and the exchange rate used in this study was 1:29. (+ − percentage) indicated the percent change as compared with the previous year.

To elucidate the nuanced patterns of drug prescription across various categories, we further subdivided the drug groups into pharmacological subgroups (3rd level) based on the ATC code. Table 3 presents the top 10 drug subgroups. Because the use of medications was much higher in OPD than in IPD, we mainly focused on OPD medications at these hospital levels. Within medical centers, the highest OPD drug expenditure was observed for L01X (other antineoplastic agents) ($440.92 million/year (median) with 23.98% incremental in 2020 and 21.44% incremental in 2021), followed by L04A (immunosuppressants) ($262.41 million/year (median) with 8.01% incremental in 2020 and 3.00% incremental in 2021), J05A (direct-acting antivirals) ($174.61 million/year (median) with 16.39% decremental in 2020 and 12.56% decremental in 2021), and A16A (other alimentary tract and metabolism products) ($125.54 million/year (median) with 10.53% incremental in 2020 and 8.14% incremental in 2021). The drug expenditure pattern in regional hospitals mirrored that of medical centers, with L01X (other antineoplastic agents) ($212.38 million/year (median) with 27.27% incremental in 2020 and 22.58% incremental in 2021) exhibiting the highest drug expenditure, followed by J05A (direct-acting antivirals) ($181.95 million/year (median) with 23.38% decremental in 2020 and 21.86% decremental in 2021), L04A (immunosuppressants) ($110.78 million/year with 9.40% incremental in 2020 and 6.48% incremental in 2021), and A10B (blood glucose-lowering drugs, excluding insulin) ($89.81 million/year (median) with 0.97% decremental in 2020 and 0.79% decremental in 2021). District hospitals allocated their highest drug expenditure to L01X (other antineoplastic agents) ($36.91 million/year (median) with 71.68% incremental in 2020 and 68.64% incremental in 2021), followed by A10B (blood glucose-lowering drugs, excluding insulin) ($57.28 million/year (median) with 6.78% incremental in 2020 and 5.73% incremental in 2021), J05A (direct-acting antivirals) ($64.88 million/year (median) with 12.70% decremental in 2020 and 29.67% decremental in 2021), and C10A (lipid-modifying agents, plain) ($35.05 million/year (median) with 5.83% incremental in 2020 and 4.58% incremental in 2021). In clinics, A10B (blood glucose-lowering drugs, excluding insulin) ($70.49 million/year (median) with 11.76% incremental in 2020 and 10.00% incremental in 2021) showcased the highest drug expenditure, followed by C10A (lipid-modifying agents, plain) ($55.93 million/year (median) with 9.57% incremental in 2020 and 9.11% incremental in 2021), C09D (angiotensin II antagonists, combinations) ($37.43 million/year (median) with 12.17% incremental in 2020 and $13.48% incremental in 2021), and N06A (psycho-analeptics) ($25.80 million/year (median) with 13.53% incremental in 2020 and 6.87% incremental in 2021). Table 3 also presents the order and differences in drug expenditures for each medical facility type.

**Table 3:** Drug expenditure among different level of hospitals based on the top 10 highest three digital Anatomical Therapeutic Chemical code.

	IPD			OPD		
	2019	2020	2021	2019	2020	2021
(A) Medical center						
L01X: other antineoplastic agents	82.93	101.25 (+22.10%)	98.18 (−3.14%)	355.64	440.92 (+23.98%)	535.44 (+21.44%)
L04A: Immunosuppressants	7.50	8.32 (+10.94%)	8.40 (+0.98%)	242.95	262.41 (+8.01%)	270.29 (+3.00%)
J05A: Direct Acting Antivirals	5.99	6.37 (+6.30%)	7.34 (+15.20%)	208.85	174.61 (−16.39%)	152.69 (−12.56%)
A16A: other alimentary tract and metabolism products	24.52	23.35 (−4.78%)	18.50 (−20.78%)	113.58	125.54 (+10.53%)	135.76 (+8.14%)
B02B: Vitamin k and other hemostatics	12.59	10.72 (−14.87%)	6.14 (−42.74%)	69.64	78.07 (+12.10%)	85.80 (+9.91%)
L01B: antimetabolites	20.07	20.04 (−0.15%)	18.28 (−8.80%)	59.43	58.23 (−2.02%)	57.47 (−1.30%)
J01D: Other beta-lactam antibacterials	63.57	66.75 (+5.00%)	69.31 (+3.83%)	5.52	4.44 (−19.46%)	4.37 (−1.75%)
B01A: Antithrombotic agents	6.24	6.22 (−0.41%)	6.30 (+1.30%)	64.85	66.29 (+2.21%)	66.92 (+0.95%)
A10B: Blood glucose lowering drugs, excl. Insulins	0.84	0.90 (+6.68%)	0.99 (+9.74%)	64.89	63.06 (−2.83%)	62.97 (−0.14%)
C10A: lipid modifying agents, plain	0.74	0.75 (+2.52%)	0.75 (−0.98%)	52.77	51.17 (−3.03%)	49.53 (−3.19%)
(B) Regional hospital						
L01X: other antineoplastic agents	68.7	80.03 (+16.48%)	74.68 (−6.68%)	166.88	212.38 (+27.27%)	260.35 (+22.58%)
J05A: Direct Acting Antivirals	2.74	2.88 (+5.14%)	2.87 (−0.14%)	237.48	181.95 (−23.38%)	142.17 (−21.86%)
L04A: Immunosuppressants	1.09	1.32 (+20.80%)	1.86 (+40.46%)	101.26	110.78 (+9.40%)	117.95 (+6.48%)
A10B: Blood glucose lowering drugs, excl. Insulins	1.19	1.21 (+1.80%)	1.25 (+2.87%)	90.68	89.81 (−0.97%)	89.10 (−0.79%)
N05A: Antipsychotics	12.01	11.97 (−0.32%)	12.08 (+0.85%)	59.59	62.23 (+4.42%)	63.86 (+2.63%)
B01A: Antithrombotic agents	5.73	5.79 (+1.03%)	5.99 (+3.39%)	64.59	65.30 (+1.10%)	65.88 (+0.89%)
J01D: Other beta-lactam antibacterials	58.95	60.25 (+2.21%)	60.19 (−0.11%)	4.24	3.77 (−11.01%)	3.65 (−3.24%)
C10A: lipid modifying agents, plain	0.82	0.84 (+2.53%)	0.82 (−2.88%)	62.94	61.82 (−1.77%)	60.14 (−2.72%)
L01B: antimetabolites	15.33	14.34 (−6.48%)	13.47 (−6.04%)	30.80	30.68 (−0.39%)	32.01 (+4.35%)
B02B: Vitamin k and other hemostatics	2.11	2.02 (−4.03%)	2.18 (+7.71%)	39.08	41.39 (+5.90%)	38.67 (−6.56%)
(C) District hospital						
L01X: other antineoplastic agents	6.80	11.18 (+64.32%)	13.92 (+24.54%)	21.50	36.91 (+71.68%)	62.25 (+68.64%)
A10B: Blood glucose lowering drugs, excl. Insulins	0.64	0.70 (+10.48%)	0.77 (+9.05%)	53.64	57.28 (+6.78%)	60.56 (+5.73%)
J05A: Direct Acting Antivirals	0.44	0.78 (+77.88%)	1.34 (+73.39%)	74.32	64.88 (−12.70%)	45.63 (−29.67%)
N05A: Antipsychotics	11.31	11.59 (+2.45%)	11.83 (+2.13%)	25.15	27.00 (+7.39%)	28.79 (+6.62%)
C10A: lipid modifying agents, plain	0.31	0.34 (+9.71%)	0.38 (+10.44%)	33.12	35.05 (+5.83%)	36.66 (+4.58%)
L04A: Immunosuppressants	0.04	0.38 (+861.05%)	0.71 (+85.49%)	25.63	30.43 (+18.70%)	35.05 (+15.17%)
J01D: Other beta-lactam antibacterials	25.92	27.55 (+6.29%)	30.28 (+9.91)	2.02	1.94 (−3.95%)	2.02 (+4.15%)
B01A: Antithrombotic agents	1.18	1.38 (+16.66%)	1.5 (+9.27%)	24.43	27.27 (+11.66%)	29.73 (+9.00%)
C09D: Angiotensin II antagonists, combinations	0.34	0.36 (+6.10%)	0.40 (+10.88%)	24.59	25.93 (+5.44%)	26.83 (+3.47%)
A10A: Insulins and analogs	0.93	0.97 (+5.18%)	0.99 (+1.32%)	17.13	17.72 (+3.42%)	18.09 (+2.11%)
(D) Clinics/others						
A10B: Blood glucose lowering drugs, excl. Insulins	0.00	0.00 (−64.39%)	0.00 (−1.090%)	63.07	70.49 (+11.76%)	77.54 (+10.00%)
C10A: lipid modifying agents, plain	0.00	0.00 (−100.00%)	0.00 (−)	51.05	55.93 (+9.57%)	61.03 (+9.11%)
C09D: Angiotensin II antagonists, combinations	0.00	0.00 (−)	0.00 (−)	33.37	37.43 (+12.17%)	42.47 (+13.48%)
N06A: Psychoanaleptics	0.00	0.00 (+0.00%)	0.00 (+275.00%)	22.72	25.80 (+13.53%)	27.57 (+6.87%)
C09C: Angiotensin II receptor blockers (ARBs), plain	0.00	0.00 (+32.05%)	0.00 (−100.00%)	23.96	25.31 (+5.63%)	25.70 (+1.52%)
J05A: Direct Acting Antivirals	0.00	0.00 (+515.38%)	0.00 (−85.68%)	28.14	26.64 (−5.32%)	21.79 (−18.22%)
C08C: Selective calcium channel blockers with mainly vascular effects	0.00	0.00 (+22.50%)	0.00 (31.42%)	20.11	18.96 (−5.75%)	17.63 (−6.97%)
A10A: Insulins and analogs	0.00	0.00 (−69.64%)	0.00 (−100.00%)	12.42	13.93 (−12.12%)	14.92 (+7.11%)
N05A: Antipsychotics	0.00	0.00 (+13.70%)	0.00 (+32.16%)	11.16	12.76 (+14.36%)	13.90 (+8.92%)
N05B: Anxiolytics	0.00	0.00 (−32.26%)	0.00 (−38.09%)	10.23	11.16 (+9.07%)	11.87 (−6.30%)

Hospital levels were categorized into four types: medical centers, regional hospitals, district hospitals, and clinics/others. The top 10 prescription ATC categories for different hospital levels were identified based on the annual sum cost of prescription drugs belonging to the same 3rd ATC level (pharmacological subgroup, form A01A to V10X), and the drug expenditure was indicated by million USD and the exchange rate used in this study was 1:29. (+ − percentage) indicated the percent change as compared with the previous year.

Furthermore, we examined OPD drug prescriptions for the highest drug expenditure item within the top 10 drug subgroups. In medical centers, osimertinib (L01XE35) recorded the highest drug expenditure at $44.20 million/year (median) with 92.75% incremental in 2021, followed by coagulation factor VIII (B02BD02) at $49.22 million/year (median), agalsidase alfa (A16AB03) at $31.55 million/year (median), and emtricitabine/tenofovir alafenamide/bictegravir (J05AR20) at $24.55 million/year (median). Regional hospitals showed the highest drug expenditure on antivirals for the treatment of Hepatitis C virus (HCV) infections (J05AP) at $87.85 million/year (median) with 37.25% decrease in 2020 and 42.85% decrease in 2021, followed by osimertinib (L01XE35) at $16.88 million/year (median), coagulation factor VIII (B02BD02) at $29.13 million/year (median), and clopidogrel (B01AC04) at $26.30 million/year (median). District hospitals exhibited the highest drug expenditure on antivirals for the treatment of HCV infections (J05AP) at $50.21 million/year (median) with an 18.12% decrease in 2020 and a 43.25% decrease in 2021, followed by valsartan and amlodipine (C09DB01) at $12.41 million/year (median), atorvastatin (C10AA05) at $10.68 million/year (median), and clopidogrel (B01AC04) at $9.90 million/year (median). Clinics saw the highest drug expenditure on valsartan and amlodipine (C09DB01) at $22.92 million/year (median) with 2.00% incremental in 2020 and 15.67% incremental in 2021, followed by atorvastatin (C10AA05) at $24.62 million/year (median), amlodipine (C08CA01) at $15.27 million/year (median), and antivirals for the treatment of HCV infections (J05AP) at $18.48 million/year (median). Because drug expenditure may be influenced by changes in prescription volume or other factors, we also analyzed the prescription volume for these highest drug expenditure OPD drugs. In general, the drug expenditure trend was aligned with the drug prescription volume trend (Table 4).

**Table 4:** Drug expenditure among different levels of hospitals based on the top 10 drugs.

	Drug expenditure			Drug prescription volume		
	2019	2020	2021	2019	2020	2021
(A) Medical center						
L01XE35 Osimertinib	0.00	44.2 (−)	92.75 (+109.83%)	0.00	331,689 (−)	732,934 (+120.97%)
J05AR20 emtricitabine, tenofovir alafenamide, and bictegravir	1.66	24.55 (+1381.18%)	33.77 (+37.56%)	109,227	1,618,064 (+1381.38%)	2,225,940 (+37.57%)
A16AB04 agalsidase beta	25.40	31.55 (+24.20%)	36.68 (+16.28%)	4,180	5,220 (+24.87%)	6,072 (+16.33%)
B02BD02 coagulation factor VIII*	45.98	49.22 (+7.03%)	52.18 (+6.01%)	56,707,348	64,633,208 (+13.98%)	69,327,984 (+7.26%)
L01BA04 pemetrexed	32.96	31.96 (−3.02%)	30.23 (−5.42%)	67,516	66,067 (−2.15%)	64,935 (−1.71%)
B01AC04 clopidogrel	23.25	22.23 (−4.40%)	21.31 (−4.13%)	18,388,580	18,090,532 (−1.62%)	18,007,160 (−0.46%)
J01DH02 meropenem	20.84	21.48 (+3.06%)	22.01 (+2.49%)	1,860,415	1,952,166 (+4.93%)	2,048,519 (+4.94%)
A10BH05 linagliptin	7.33	7.21 (−1.63%)	6.90 (−4.39%)	11,595,084	11,625,201 (+0.26%)	11,409,022 (−1.86%)
C10AA05 atorvastatin	20.57	20.78 (+1.02%)	20.38 (−1.89%)	30,027,076	31,371,008 (+4.48%)	32,286,872 (+2.92%)
N05AX13 paliperidone	11.99	12.83 (+7.00%)	13.47 (+5.02%)	890,496	862,341 (−3.16%)	823,276 (−4.53%)
(B) Regional hospital						
J05AP Antivirals for treatment of HCV infections	140.00	87.85 (−37.25%)	50.20 (−42.85%)	2,004,142	1,425,644 (−28.87%)	851,980 (−40.24%)
L01XE35 Osimertinib	0.00	16.88 (−)	37.58 (+122.59%)	0.00	91,052 (−)	203,669 (+123.68%)
L04AB04 adalimumab	16.82	18.38 (+9.28%)	18.48 (+0.50%)	35,461	39,261 (+10.72%)	40,072 (+2.07%)
A10BH05 linagliptin	10.55	10.69 (+1.41%)	10.43 (−2.46%)	16,741,889	17,294,452 (+3.30%)	17,339,176 (+0.26%)
N05AX13 paliperidone	14.63	16.13 (+10.27%)	17.81 (+10.39%)	1,430,455	1,478,434 (+3.35%)	1,559,162 (+5.46%)
B01AC04 clopidogrel	26.30	26.33 (+0.10%)	26.12 (−0.80%)	20,949,466	21,563,652 (+2.93%)	22,200,594 (+2.95%)
C10AA05 atorvastatin	20.67	21.04 (+1.82%)	20.42 (−2.98%)	32,810,202	34,583,672 (+5.41%)	35,024,112 (+1.27%)
J01DH02 meropenem	11.43	12.47 (+9.07%)	11.99 (−3.83%)	991,389	1,092,883 (+10.24%)	1,079,449 (−1.23%)
L01BA04 pemetrexed	17.76	15.89 (−10.53%)	15.74 (−0.94%)	38,506	35,571 (−7.62%)	36,493 (+2.59%)
B02BD02 coagulation factor VIII*	29.13	30.86 (+5.95%)	26.66 (−13.59%)	37,170,968	40,640,860 (+9.33%)	35,691,760 (−12.18%)
(C) District hospital						
J05AP Antivirals for treatment of HCV infections	61.33	50.21 (−18.12%)	28.50 (−43.25%)	68,867	14,736 (−78.60%)	1,001 (−93.21%)
A10BD08 metformin and vildagliptin	6.13	6.21 (+1.38%)	6.22 (+0.10%)	13,647,774	15,723,724 (+15.21%)	16,282,984 (+3.56%)
N05AH04 quetiapine	8.30	8.66 (+4.40%)	8.92 (+2.95%)	15,264,334	16,776,926 (+9.91%)	18,258,974 (+8.83%)
C10AA05 atorvastatin	9.89	10.68 (+7.98%)	11.37 (+6.52%)	16,389,333	18,177,116 (+10.91%)	20,245,444 (+11.38%)
L01XE35 Osimertinib	0.00	2.63 (−)	10.34 (+293.61%)	0	13,752 (−)	53,156 (+286.53%)
J01DH02 meropenem	5.77	6.74 (+16.83%)	7.35 (+8.97%)	486,777	562,524 (+15.56%)	623,444 (+10.83%)
L04AB06 golimumab	4.71	5.93 (+25.78%)	6.84 (+15.40%)	4,410	5,568 (+26.26%)	6,447 (+15.79%)
B01AC04 clopidogrel	9.05	9.90 (+9.43%)	10.70 (+8.08%)	7,203,215	8,104,149 (+12.51%)	9,082,635 (+12.07%)
C09DB01 valsartan and amlodipine	12.07	12.41 (+2.80%)	12.72 (+2.53%)	24,877,696	26,184,508 (+5.25%)	27,730,066 (+5.90%)
B02BD02 coagulation factor VIII*	12.56	9.49 (−24.41%)	8.35 (−12.06%)	15,739,778	12,233,016 (−22.28%)	10,726,518 (−12.32%)
(D) Clinics/others						
A10BA02 metformin	9.17	9.36 (+2.01%)	9.56 (+2.18%)	164,774,672	169,135,232 (+2.65%)	174,504,752 (+3.17%)
C10AA05 atorvastatin	23.00	24.62 (+7.06%)	25.92 (+5.25%)	54,256,536	60,777,800 (+12.02%)	68,141,536 (+12.12%)
C09DB01 valsartan and amlodipine	22.47	22.92 (+2.00%)	26.51 (+15.67%)	60,095,632	63,557,436 (+5.76%)	74,617,856 (+17.40%)
J05AP Antivirals for treatment of HCV infections	20.22	18.48 (−8.62%)	13.52 (−26.84%)	328,573	332,103 (+1.07%)	253,191 (−23.76%)
C09CA08 Olmesartan	4.55	7.79 (+71.14%)	8.35 (+7.14%)	9,072,812	14,877,530 (+63.98%)	23,757,904 (+59.68%)
N06AB10 escitalopram	7.24	7.86 (+8.54%)	8.12 (+3.35%)	22,069,612	25,790,508 (+16.86%)	29,574,170 (+14.67%)
C08CA01 amlodipine	16.21	15.27 (−5.76%)	14.18 (−7.16%)	120,464,400	118,787,056 (−1.36%)	116,470,672 (−1.95%)
M01AB05 diclofenac	3.92	3.28 (−16.16%)	2.92 (−11.12%)	158,280,864	129,689,904 (−18.06%)	112,476,392 (−13.27%)
A10AE04 insulin glargine	7.62	8.22 (+7.78%)	8.49 (+3.35%)	524,694	557,997 (+6.35%)	582,344 (+4.36%)
R06AE09 levocetirizine	2.65	2.65 (+0.26%)	2.55 (−3.88%)	43,684,112	44,361,760 (+1.55%)	43,808,084 (−1.25%)

Hospital levels were categorized into four types: medical centers, regional hospitals, district hospitals, and clinics/others. ^*^The drug prescription volume unit used in this drug is I.U. The top 10 prescription ATC categories for different hospital levels were identified based on the annual sum cost of prescription drugs belonging to the same 5th ATC level (chemical substance, form A01AA01 to V10XX05), and the drug expenditure was indicated by million USD and the exchange rate used in this study was 1:29. (+ − percentage) indicated the percent change as compared with the previous year. The drug prescription volume trend for these highest drug expenditure items among the top 10 drug subgroups across different hospital levels was measured using the sum prescription quantity.

## Discussion

This study explored variations in drug expenditure across different hospital types and drug categories in Taiwan. We observed a consistent increase in drug expenditure across all hospital types in Taiwan in 2020, similar to the patterns observed in 2018 or 2019, based on a comprehensive analysis of a nationwide health insurance database (9). However, in 2021, drug expenditure across all hospital types in Taiwan decreased (Table 1). This decline aligns with findings from other studies that reported a decrease in the number of notifiable infectious diseases during the COVID-19 pandemic (12). Notably, antitumor and immunological agents accounted for the highest drug expenditures in medical centers, regional hospitals, and district hospitals. In contrast, cardiac and hypertension agents were the top drug expenditure categories in clinics and other primary care settings (Table 2). These results support the Ministry of Health and Welfare’s policy of increasing reimbursement service fees and incentives to encourage medical centers and regional hospitals to focus on severe illnesses and emergency care (13). During 2019–2021, annual OPD drug expenditures for other antineoplastic agents in medical centers, regional hospitals, and district hospitals increased despite the COVID-19 outbreak in Taiwan (Table 3). Similarly, annual drug expenditures for diabetes mellitus (metformin), hyperlipidemia (atorvastatin), and hypertension (valsartan and amlodipine) in clinics and other primary care settings also increased (Table 4).

Taiwan implemented various measures to prevent COVID-19 outbreaks (14), including strict border controls for rigorous screening of incoming travelers, mandatory quarantine, and implementation of testing protocols during 2020–2022 (15). The country employed extensive contact tracing to identify and strictly enforce those who tested positive or had potential exposure (15). In 2020, Taiwan recorded only 56 locally acquired confirmed cases of COVID-19 after testing 126,987 individuals (16, 17). Drug expenditure increased slightly during this period. In 2021, the Central Epidemic Command Center raised the epidemic warning to level 3 nationwide in response to a surge in indigenous COVID-19 cases (18). Taiwan promptly contained this large outbreak at approximately 15,000 cases. Individuals conducted self-initiated prevention by reducing social activity or outpatient/medical consultations. Previous studies showed that drug expenditure in Taiwan increased in all hospitals between 2016 and 2018 (9). Compared with that during the COVID-19 pandemic, drug expenditure decreased in 2021 (Table 1). COVID-19 caused self-initiated decreases in outpatient/medical consultations, which may be one of the major causes; however, the government’s policy to control drug expenditure by allocating a fixed amount of money for a predetermined set of services (Global Budgeting System) may also have contributed to the decrease in drug expenditure in 2021 (19).

In the context of COVID-19 treatment, healthcare providers have been prescribing antiviral medications and other experimental drugs under emergency use authorization or clinical trials (20). This has changed the prescribing practices for these specific conditions. Unlike other countries, the incidence of COVID-19 infection in Taiwan was low (56 locally acquired confirmed cases in 2020 and 15,000 cases in 2021). The impact of anti-infectives for systemic use was not significant. We observed that the drug expenditure for anti-infectives for systemic use decreased in 2020 and 2021 (Table 3). Among these anti-infectives for systemic agents, using direct-acting antivirals (J05A, DAA) showed a dramatic reduction in 2021, which may have contributed to the decreased drug expenditure (Table 4). DAA agents were part of the reimbursement program in 2017, and their prescriptions increased over time (9). DAA agents strategically intervene at various stages of the HCV replication life cycle, demonstrating remarkable efficacy with a brief treatment duration. Comprehensive studies have consistently indicated that DAA therapy achieves a cure rate exceeding 90%, boasting a favorable safety profile and potentially yielding unforeseen advantages for patients (21, 22). The Ministry of Health and Welfare removed treatment restrictions based on the fibrosis stage in 2019, leading to higher drug expenditures than in 2018 (9). The number of DAA-treated patients increased from 9,500 in 2017 to 46,000 in 2019 (23). Given that DAA therapy achieves a cure rate exceeding 90%, the number of patients with HCV who seek DAA treatment may have decreased; thus, drug expenditure may have decreased in 2020 and 2021.

Although analysis of NHIRD data has yielded various advantages, inherent limitations persist because of the NHIRD design, which lacks blind randomization. First, data gaps exist concerning self-payment-funded medications due to the NHIRD’s specific objectives. Disease severity remains challenging to quantify. Second, our study relies solely on the claim system, which introduces potential inaccuracies from coding errors and misclassifications. Third, the generalizability of our results may be limited by differences in healthcare systems, demographics, and lifestyle factors across regions or countries. Finally, the extensive time required by the NHI to compile and release annual data precluded the inclusion of the most recent patients in our study, emphasizing the need for additional data for long-term observations.

## Conclusion

Our findings revealed a consistent rise in annual drug expenditures across all hospitals from 2019 to 2020 but a decrease from 2020 to 2021. This effect may be caused by the number of locally acquired confirmed cases of COVID-19 in 2020 (only 56), and an outbreak of approximately 15,000 cases occurred in 2021. Notably, antitumor and immunological agents had the highest drug expenditures at medical centers, regional hospitals, and district hospitals. In contrast, cardiac and hypertension drugs were the highest expenditure categories in clinics. While drug expenditure for anti-infectives for systemic use decreased in 2020 (−15.9%) but slightly increased in 2021 (0.6%) in the United States (24, 25), in Taiwan, drug expenditure for anti-infectives for systemic use decreased in 2020 and 2021 across all hospital types, and the decrease in the use of direct-acting antivirals (J05A, DAA) may be one of the major causes.

## Data Availability

The data analyzed in this study is subject to the following licenses/restrictions: Taiwan national claim data sets are available through formal application to Applied Health Research Data Integration Service provided by the BNHI of Taiwan by provide detail research proposal. Restrictions applied to these data, which were used under license for our study, and so are not publicly available for duplication. Further data analysis may be requested after discussion with authors. Requests to access these datasets should be directed to stpeicih@mohw.gov.tw.
